# Pilot-Scale Ensilaging of Herring Filleting Co-Products and Subsequent Separation of Fish Oil and Protein Hydrolysates

**DOI:** 10.1007/s11947-022-02870-9

**Published:** 2022-07-19

**Authors:** Mursalin Sajib, João P. Trigo, Mehdi Abdollahi, Ingrid Undeland

**Affiliations:** Food and Nutrition Science, Department of Biology and Biological Engineering, Chalmers University of Technology, 41296 Gothenburg, Sweden

**Keywords:** Herring, By-products, Valorization, Ensilaging, Silage, Pilot scale

## Abstract

In this study, ensilaging of herring (*Clupea harengus*) filleting co-products was taken from lab-scale to pilot scale (1500 L) while monitoring the protein degree of hydrolysis (DH) and lipid oxidation. Subsequently, the possibility of recovering fish oil and protein hydrolysates using batch centrifugation at different g-forces/times was investigated. Around 38% DH was recorded after 2-day pilot-scale ensilaging of herring co-products at ambient temperature (i.e., ~ 22 °C), which was similar to the DH found in lab-scale (40% after 2 days; 22 °C). The lipid oxidation marker 2-thiobarbituric acid reactive substances (TBARS) reached 20 µmole TBARS/kg silage after 2-day ensilaging. Centrifugation of the silage at 3000–8500 × g for 2–20 min revealed successful separation into fish oil and protein hydrolysates. Heat-treating the silage (85 °C; 30 min) prior to centrifugation resulted in significantly higher oil and hydrolysates recoveries; the same being true for increased g-force. At 8500 × g, the recovery of oil and hydrolysates were 9.7 and 53.0% *w*/*w*, respectively, from heat-treated silage, while recoveries were 4.1 and 48.1% *w*/*w*, respectively, from non-heat treated silage. At 4500 × g, being a more scalable approach, corresponding numbers were 8.2 and 47.1% (*w*/*w*) as well as 2.0 and 40.2% (*w*/*w*). The recovered fish oil contained 8% EPA and 11% DHA of total fatty acids. Free fatty acids (FFA), peroxide value (PV), p-anisidine value (p-AV), and total oxidation (TOTOX) values of oils were in the range of 4–7% (FFA), 3.6–3.7 meq/kg oil (PV), 2.5–4.0 (p-AV), and 9.9–11.1 (TOTOX), respectively, which were within the acceptable limits for human consumption specified by the GOED voluntary monograph. The recovered protein hydrolysates contained peptides in the molecular weight range 0.3–6 kDa (~ 37%) and 11–34 kDa (~ 63%). Also, the remaining solids contained 15–17% (*w*/*w*) protein, having 44–45% essential amino acids. Overall, the results suggest that herring co-product silage is a valuable source of fish oil and protein hydrolysates, paving the way for ensilaging based-biorefining of herring co-products into multiple products.

**Supplementary Information:**

The online version contains supplementary material available at 10.1007/s11947-022-02870-9.

## Introduction

To meet the growing demand for fish, and especially for convenience products, around 70% of all caught/harvested fish is processed before final sale, generating around 20–80% co-products depending on the type of fish and level of processing (Ghaly et al., 2013). For example, in Sweden, around 20,000 tons of herring are processed annually into convenience products (e.g., fillets), generating around 12,000 tons of co-products. These co-products have traditionally been exported for lower value applications like mink feed or fishmeal production, which can be questioned considering, e.g., environmental impacts of transportation and high energy use in fish meal/fish oil and subsequent feed productions (Hilmarsdottir et al., 2020, 2021, 2022). Here, ensilaging—endogenous protease-mediated autolysis under acidic conditions—could provide a cost-efficient and “green” process option to valorize these co-products into protein hydrolysates (Olsen & Toppe, 2017), traditionally known as silage, which is also easily implementable locally at fish processing plants.

Lab-scale ensilaging of herring filleting co-products has previously been reported, e.g., by our team (Sajib et al., 2020, 2022), but also by, e.g., van’ t Land et al. (2017). Briefly, ensilaging can be performed by adding 2.5% *v/w* formic acid (85% purity) to minced herring co-products to lower the pH below 4.0, and the protein degree of hydrolysis (DH) can then be tuned by terminating the autolysis after a specific time. Based on our earlier findings, most changes in DH appear within 1–3 days of ensilaging, and around 40% DH was achieved after 2 days of ensilaging at 22 °C with continuous stirring. Although susceptible to lipid oxidation (Sajib & Undeland, 2020), the silage could however, be stabilized during its production, heat-treatment, and storage by adding rosemary (*Rosmarinus officinalis* L.) extract-based antioxidants (Sajib et al., 2022). For example, BORDANTIX W/S (0.25% *w/w*), a commercial rosemary extract-based antioxidant mixture, lowered 2-thiobarbituric acid reactive substances (TBARS) formation by 91% after 7 days of ensilaging at 22 °C, compared to the control. However, translating lab-scale data into pilot-scale processing is always challenging (de la Fuente et al., 2020) due to, e.g., variations in raw material volume, type of ensilaging container (specifically its volume to surface ratio), chopper, and stirrer. To date, very little is reported about the DH and lipid oxidation development during pilot-scale ensilaging of fish filleting co-products (Arason et al., 1990; Young & Dunn, 1975).

There are strong reasons to believe that herring co-product silage can be a good source of health-promoting long-chain (LC) omega-3 polyunsaturated fatty acid (PUFA)-rich fish oil as high levels of eicosapentaenoic acid (EPA) and docosahexaenoic acid (DHA) are found in the head, backbone, belly flap, and intestines of this species (Wu et al., 2021). EPA and DHA have been reported to be negatively associated with cardiovascular disease (CVD) and CVD risk factors like elevated plasma lipids and high blood pressure (Endo & Arita, 2016; Hirafuji et al., 2003; Jump et al., 2012). These fatty acids are also suggested in potential adjuvant therapeutic strategies for COVID-19 patients (Darwesh et al., 2020; Szabó et al., 2020; Weill et al., 2020). The global market value for omega-3 PUFAs was USD 2.49 billion in 2019 and is expected to grow at a compound annual growth rate (CAGR) of 7% over the period 2020–2027 (Oliver et al., 2020). However, the traditional marine omega-3 PUFA sources are exhausted (Oliver et al., 2020); thus increasing the use of co-products from fish filleting are viable option to produce omega-3 PUFAs for the food and feed markets. Silage oil is an interesting alternative to traditionally produced oil as long transport of the non-stabilized co-product raw materials to a meal/oil plant is not needed, and since less heat is used—only in the silage sterilization/hygienization step. This would imply lower oxidative losses of LC omega-3 PUFA and less consumption of endogenous antioxidants, yielding a higher quality oil. To date, however, very little is known about the recovery of omega-3 PUFA-rich oils from herring co-product silage.

Further to LC omega-3 PUFA, herring co-product silage indeed also contains short-chain peptides in the molecular weight range of 0.2–4.7 kDa (Sajib et al., 2020), and thus may possess numerous bioactive properties. For example, it has been reported that short-chain peptides—i.e., with 2–20 amino acids (Meisel & FitzGerald, 2003) and < 6 kDa sizes (Sun et al., 2004)—may possess, e.g., anti-oxidative, antihypertensive, and anti-inflammatory activities upon intake (Melgosa et al., 2021; Rodrigues et al., 2021; Sarmadi & Ismail, 2010). Besides bioactive properties, marine protein hydrolysates could also provide cryoprotective and antioxidative effects in frozen seafood products (Nikoo et al., 2016). The global market value for fish protein hydrolysates was USD 407 million in 2019, and is expected to grow at a CAGR of 4.7% over the period 2019–2027 (Markets, 2021); here silage protein hydrolysates could provide a cost-efficient source to this growing market.

To the best of our knowledge, very little work has been done on the separation of herring co-product silage into fish oil and protein hydrolysates. Özyurt et al. (2018) reported fish oil recovery from sea bass (*Dicentrarchus labrax*) by-product silage by first heating the silage to 50 °C using a water bath followed by centrifugation at 7000 g for 20 min. Several other authors have reported the separation of fish protein hydrolysates, made by exogenously added proteases, into different phases by first heat-treating the hydrolysates (85–100 °C) to inactivate enzymes, followed by centrifugation at 1700–10,000 g for 10–30 min (Liaset et al., 2002; Liu et al., 2020a, b; Mbatia et al., 2010; Santos et al., 2015; Šližyte et al., 2005a, b, 2017). However, none of these authors investigated the main and interaction effects of separation settings, e.g., heat-treatment, g-force, and centrifugation time on oil/hydrolysate recovery yields.

The aims of this study were to (i) upscale ensilaging of herring filleting co-products from lab-scale to 1500 L-scale while monitoring the DH and lipid oxidation, and (ii) investigate the effect of centrifugation time, g-force, and heat treatment on lab-scale separation of silage into fish oil and protein hydrolysates. The results of this study can thus aid valorization of herring co-product raw materials, which currently have a low market value, into multiple high-value products.

## Material and Methods

### Materials

**Fig. 1:**
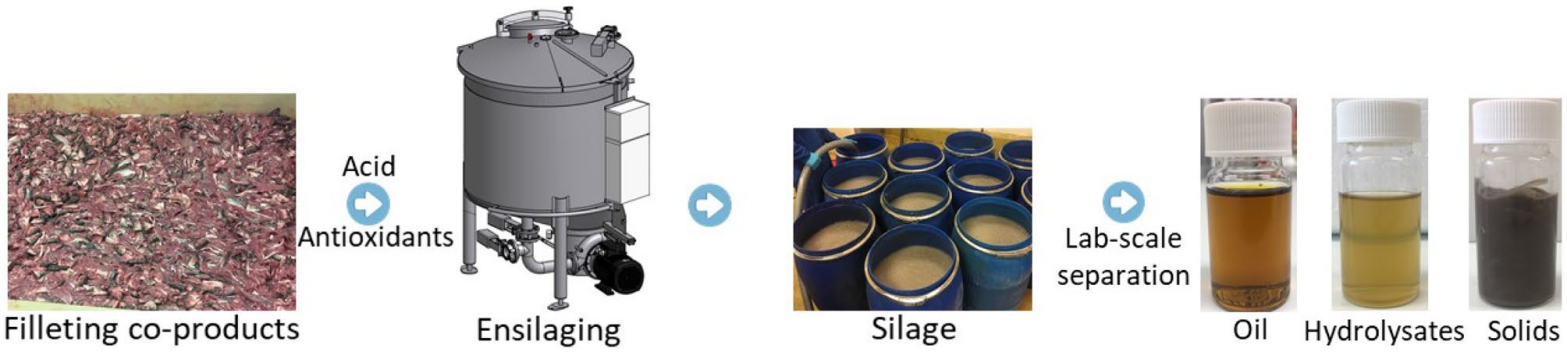
Pilot-scale ensilaging of herring filleting co-products and subsequent lab-scale separation of silage into fish oil, protein hydrolysates, and solids. The silage was separated into oil, protein hydrolysates, and solids by centrifugation of the silage at 3000–8500 × g for 2–20 min

Herring filleting co-products—consisting of a mix of heads, frames, tails, skins, guts, and other intestinal organs—were kindly provided by Sweden Pelagic AB (Ellös, Sweden). The co-products were from herring filleted on the 30th of September 2020 and collected immediately after filleting and ensilaged as described later. A 2.0 m^3^-capacity semi-automated BioChop ensilaging tank (Fig. 1, Landia A/S, Lem, Denmark), equipped with a BioChop pump for chopping, mixing, and removal of the product, as well as a heating jacket for heating/cooling through hot/cold water circulation, was used for pilot-scale ensilaging. Formic acid (85% purity) was purchased from Fisher Scientific (Göteborg, Sweden), and the antioxidants used were BORDANTIX LIQUID W/S and BORDANTIX LIQUID O/S (EVESA, Cádiz, Spain).

### Pilot-Scale Ensilaging of Herring Filleting Co-Products

Around 1420 kg of herring filleting co-products were first chopped at 1000 rpm for 15 min, followed by the addition of 4.0 kg of BORDANTIX W/S and 4.0 kg of BORDANTIX O/S (thus reaching 0.56% *w*/*w* antioxidant concentration) and mixed at 1000 rpm for 15 min (Fig. 1). The co-products were then ensilaged by adding 35.50 L of formic acid (85% purity; 2.5% *v*/*w* formic acid to co-product ratio) (Sajib et al., 2020). The mixture was then mixed at 1000 rpm for 1 h, followed by intermittent mixing every third hour at 200 rpm for 30 min. Ensilaging was performed at ambient temperature (i.e., 21–22 °C), and the pH values of silages were in the range of 3.68–3.74 during the studied period. Samples were collected after 1 h, 1 day, and 2 days of ensilaging, and stored immediately at − 20 °C, whereafter they were transferred to − 80 °C. After 2 days of ensilaging, the silage was then transferred to 100-L plastic barrels or 5-L plastic storage containers and stored at 4 °C.

### Proximate Composition Analysis

The moisture content was analyzed according to the NREL protocol (NREL/TP-510–42,621) by drying the samples at 105 °C for 24 h (Sluiter et al., 2008). The crude protein content was determined by first analyzing total nitrogen content using a nitrogen analyzer (LECO) and then converting the value to protein content using nitrogen to a protein conversion factor of 5.58 (Mariotti et al., 2008). Crude lipid content was determined gravimetrically using a portion of the chloroform phase from a chloroform:methanol (2:1) extraction according to Lee et al. (1996). Ash content was determined according to NREL protocol (NREL/TP-510–42,622) by ashing the samples at 575 °C for 4 h (Sluiter et al., 2005).

### Determination of Protein Degree of Hydrolysis (DH)

DH was determined spectrophotometrically (Cary 60 UV–vis, Agilent technologies, 117 USA) using o-phthaldialdehyde (OPA) reagent according to Nielsen et al. (2001) with slight modifications as described by Sajib et al. (2020).

### Determination of Peroxide Value (PV) and 2-Thiobarbituric Acid Reactive Substances (TBARS)

Samples for PV and TBARS analyses were prepared as described by Sajib et al. (2022) using a chloroform:methanol (2:1) extraction according to Lee et al. (1996). The PV and TBARS were then analyzed spectrophotometrically (Cary 60 UV–vis, Agilent technologies, 117 USA) using the chloroform and methanol phase, respectively, according to Undeland et al. (2002) and Schmedes and Hølmer (1989), respectively.

### Lab-Scale Separation of Silage into Fish Oil and Protein Hydrolysates

Around 10 mL silage samples were transferred to 15-mL tubes; half of these samples were heat-treated at 85 °C for 30 min and then cooled down to room temperature. Thereafter, silage samples, heat treated and not heat treated, were centrifuged at either 3000, 4500, or 8500 × g for 2, 10, or 20 min at 20 °C. Phases after centrifugation were collected, weighed, and stored at − 80 °C until further use. Recovery yields were calculated based on the weight (as it is) of the collected phase after centrifugation, compared to the weight (as it is) of the silage sample used before centrifugation.

To further understand the main and interaction effects of the studied factors, i.e., heat-treatment, g-force, and centrifugation time on recovery yields, a mixed full factorial design was used from which the retrieved data set was subjected to multivariate analysis using MODDE Pro (version 12.1, Sartorius Stedim Data Analytics AB, Sweden). The yields were scaled and centered, and the model was fitted with partial least squares (PLS) regression. The coefficient plots are presented in this study, and the size of coefficients shows the change in yields when a factor varies from its medium to high level while keeping other factors at their average levels.

### Color Measurement

The color of oil samples was measured in the CIE L ∗ a ∗ b ∗ color space using a colorimeter (CR-400, Konica Minolta Sensing, Japan) as described by Abdollahi et al. (2016).

### Determination of Fatty Acid Composition

Fatty acids were analyzed as follows: samples were weighed into capped glass tubes and internal standard, 100 µg of heptadecanoic acid (100 µL of 1 mg/mL stock in toluene), was added. Three mL of chloroform:methanol (2:1 *v*/*v*) was added into the tubes followed by mixing and addition of 2.75 mL 0.9% NaCl. Samples were then mixed thoroughly and centrifuged at 3000 g for 6 min. The lower phase was evaporated to dryness after which 1 mL toluene and 1 mL 10% *v*/*v* hydrochloric acid in methanol was added to the samples. The glass tubes were capped and incubated at 60 °C for 120 min. The tubes were then allowed to cool to room temperature and 1 mL of ultra-pure water was added. Thereafter, 1 mL hexane was added, the glass tubes were mixed for 60 s and phase separation was achieved by centrifugation at 100 × g for 6 min (Heraeus multifuge 1 s). The upper phase was transferred to GC vials. An external standard was prepared from the GLC-462 fatty acid standard mix (Nu-Chek Prep, Inc.).

Samples were analyzed by using a Shimadzu TQ-8030 GC–MS/MS system consisting of a Shimadzu GC-2010 Plus gas chromatograph, Shimadzu TQ-8030 triple quadrupole mass spectrometer and a Shimadzu AOC-5000 Plus sample handling system (Shimadzu Europe GmbH, Duisberg, Germany). Data was acquired with Shimadzu GCMS Solutions software version 4.2. One milliliter of each sample was injected, and separation was done on a 30 m × 0.25 mm × 0.25 mm Zebron ZB-WAXplus column (Phenomenex, USA). The injection port temperature was set to 275 °C and the oven temperature program was as follows: initial temperature 100 °C, ramped to 205 °C at 4 °C/min followed by ramping to 230 °C at 1 °C/min and held for 5 min. The GC was operated in constant linear velocity mode set to 37.2 cm/s and septum purge flow was set to 3 mL/min. Interface and ion source temperatures were 280 and 230 °C, respectively. The autosampler was kept at 8 °C and helium was used as the carrier gas. Data was collected between 50 and 550 m/z and at single ions at 55, 74, and 87 m/z.

### Determination of Free Fatty Acids (FFA)

Oil samples were prepared according to Abdollahi and Undeland (2020), with slight modifications, and then analyzed for free fatty acids (FFA) according to a method described by Lowry and Tinsley (1976). Briefly, 200 µL of diluted fish oil samples/oleic acid standard was mixed with 1.8 mL isooctane (Marseno et al., 1998), vortexed for 10 s, followed by the addition of 400 µL cupric acetate-pyridine reagent (5% *w*/*w* cupric acetate in MQ-water; pH adjusted to 6.0 using pyridine), vortexed for 90 s, and then the absorbance of the resulting upper phase after centrifugation at 2000 × g for 10 min was read at 710 nm (Cary 60 UV–vis, Agilent technologies, 117 USA).

### Determination of P-Anisidine Value (p-AV) and Total Oxidation Value (TOTOX)

p-AV of oils was analyzed according to AOCS’s official method Cd 18–90 as described by Semb (2012) with slight modifications in the sample preparation. Briefly, 0.3 g fish oil sample was dissolved in 60 mL isooctane, vortexed to dissolve completely, and then the first absorbances of samples and the blank (i.e., isooctane) were read at 350 nm (Cary 60 UV–vis, Agilent technologies, 117 USA). Thereafter, 0.5 mL of p-anisidine reagent (0.25% *w/v* p-anisidine in glacial acetic acid) was added to 2.5 mL of samples/blank, vortexed for 30 s, followed by incubation in the dark for 10 min, and then the second absorbances of samples and the blank were read at the same wavelength as mentioned earlier, and the p-AV values were calculated according to Semb (2012). The TOTOX values were calculated from PV and p-AV values (i.e., TOTOX = 2 PV + p-AV).

### Determination of Tocopherols

Samples were prepared for tocopherols analysis by dissolving fish oil in chloroform:methanol (2:1) and then analyzed by HPLC with fluorescence detection according to a method described by Larsson and Undeland (2010).

### Microscopic Analysis of Emulsions

Microscopic investigation of emulsions, formed during centrifugation of silage, was performed as described by Abdollahi et al. (2019) using a light microscope (Axiostar Plus, Carl Zeiss Microscopy, LLC, USA) with × 40 magnification (A-Plan × 40/0.65 Ph2, Carl Zeiss Microscopy, LLC, USA), and the images were taken with a camera (Canon PowerShot G9, 12.1 Megapixels) mounted on top of the microscope with × 6 optical zoom lens.

### Analysis of Molecular Weight Distribution of Proteins and Peptides

The molecular weight distribution of soluble proteins and peptides in the hydrolysates fraction was analyzed by high-performance size exclusion chromatography (HP-SEC) according to a procedure described by Sajib et al. (2020) with slight modifications for sample preparation. Briefly, samples were diluted with the mobile phase (0.1 M sodium phosphate buffer; pH 7.5) to a protein concentration of 25 mg/mL, centrifuged at 10,000 × g for 10 min, and then the resulting supernatant was filtered (0.45 µm, fisher scientific) and used for the HP-SEC analysis. AdvanceBio SEC 300 Å Protein Standard (Agilent Technologies) was used for the analysis of molecular weights.

Molecular weight distribution was also analyzed by gel electrophoresis (SDS-PAGE). Freeze-dried samples were mixed with 5% SDS (4 mg protein/mL), and homogenized with polytron (6000 rpm, 1 min), where after protein content of the solution was measured according to Markwell et al. (1978). Samples were subsequently diluted further to a final protein concentration of 2 mg/mL. The diluted samples were mixed with tricine sample buffer (1:1 ratio) containing 2% *v*/*v* β-mercaptoethanol, followed by heating at 95 °C for 5 min in a heating block, and then centrifuged at 5000 × g for 5 min. Thereafter, 20 μg of protein from each sample as well as 5 μL of protein ladder (Precision Plus Protein Dual Xtra, 2–250 kDa, Bio-Rad) were loaded onto Tris-tricine precast mini gels (16.5%; Bio-Rad). Electrophoresis was performed at a constant voltage of 100 V (Mini Protein II unit; Bio-Rad). The gel was fixed in Coomassie Brilliant Blue R-250 destaining solution (Bio-Rad) for 60 min, followed by staining with Bio-Safe Coomassie stain (Bio-Rad) for 90 min, and then kept in MQ-water overnight to destain. A picture of the gel was taken with GelDoc Go imaging system (Bio-Rad) and then the molecular weight distribution was analyzed with Image Lab 6.1 software (Bio-Rad).

### Determination of Total Amino Acids

Samples for total amino acid analysis were prepared as described in Sajib and Undeland (2020) and then analyzed using LC/APCI-MS according to a method described by Harrysson et al. (2018).

### Statistical Analysis

One batch of silage was produced on a pilot scale, but the separation of oil/hydrolysates from the silage was done in quadruplicates (*n* = 4) at each setting. Analyses of the raw material, silage, and the fractions derived thereof were then done in triplicate (*n* = 3). Results are expressed as mean values ± standard error of the mean (SEM) from the triplicates/quadruplicates, and mean values from the replications were used in the calculations. The data sets were subjected to ANOVA analysis with Tukey’s honest significant differences (HSD) test on RStudio software (https://www.rstudio.com/), and significant differences were accepted at *p* < 0.05.

## Results and Discussion

### DH and Lipid Oxidation During Pilot-Scale Ensilaging of Herring Co-Products

**Fig. 2:**
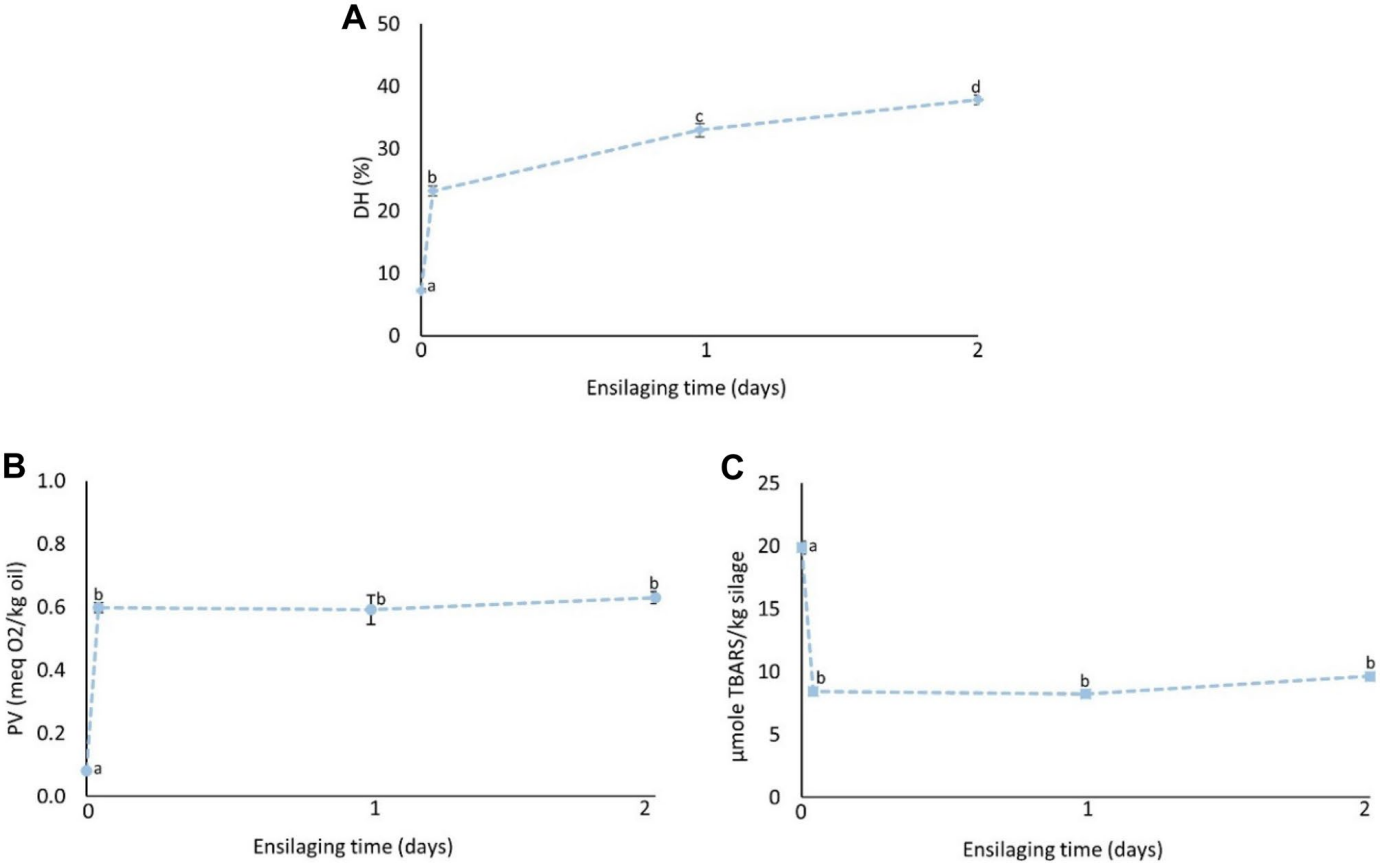
Changes in DH (**A**), PV (**B**), and TBARS (**C**) during pilot-scale ensilaging of herring co-products. Time point zero (i.e., day 0) refers to herring co-products before ensilaging. Results are expressed as mean ± SEM (*n* = 3). Different lower-case letters denote significance (*p* < 0.05) differences

The changes in DH, PV, and TBARS during pilot-scale ensilaging of herring co-products are shown in Fig. 2. The DH increased significantly (*p* < 0.05) during the studied ensilaging period and around 38% DH was recorded after 2 days at ambient temperature (i.e., 21–22 °C), which was similar to our previously reported DH value of 40% after 2-day lab-scale ensilaging at 22 °C (Sajib et al., 2020). This suggests that our lab-scale DH values well simulated what happens in pilot-scale ensilaging. There was an unexpected increase in PV already after 1 h of ensilaging, which later remained constant throughout the studied period. This could possibly be due to the inefficient mixing of antioxidants with the co-products at the beginning of the ensilaging; that is, the chopper and the sampling outlets were located at the bottom of the ensilaging tank, while the antioxidant and acid were added from the top (Fig. 1). Contrary, a high TBARS value was recorded in the herring co-product even before ensilaging, which after 1 h decreased significantly (*p* < 0.05) and remained constant at ~ 8–9 µmole TBARS/kg silage thereafter. Overall, the TBARS values recorded in this study were slightly lower than in our previously reported lab-scale ensilaging of herring co-products at 21–22 °C with 0.25–1.25% BORDANTIX W/S or BORDANTIX O/S antioxidant added; 8–9 µmole and 20–28 µmole TBARS/kg silage, respectively (Sajib et al., 2022). The lower TBARS values recorded in this study could possibly be due to the use of higher quality starting raw material as ensilaging was done on-site at the herring processing plant within 1–2 h after the generation of co-products. This stresses the advantage of local and fast valorization of raw materials with high susceptibility to oxidation to ensure a high-quality end product.

### Separation of Silage into Fish Oil and Protein Hydrolysates

**Fig. 3:**
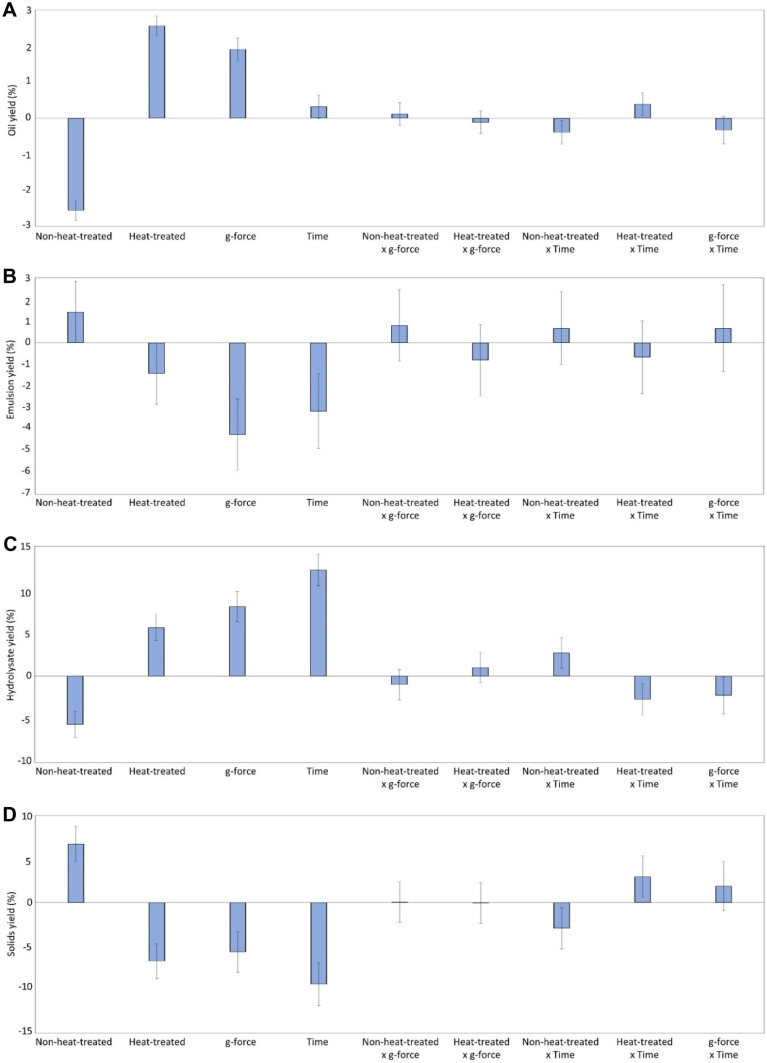
Coefficient plots showing responses of studied factors (i.e. heat-treatment, g-force, and centrifugation time) with respect to yield of oil (**A**), emulsion (**B**), hydrolysates (**C**), and solids (**D**). Responses were scaled and centered; and the size of coefficients represents the change in their respective responses when a factor varies from medium to high level, while keeping other factors at their average values

The effects of pre-heat-treating the silage at 85 °C for 30 min, centrifugation g-force, and centrifugation time on the recovery yield of oil, protein hydrolysates, and solids are shown in Fig. 3. Heat treating the silage, which can be done to inactivate enzymes and to create a microbially stable product, resulted in a significantly (*p* < 0.05) higher oil and hydrolysates recovery at all g-forces, compared to the non-heat-treated silage. Increasing the g-force had the same effect on the recovery yields while increasing the centrifugation time only increased the recovery of the protein hydrolysate phase. At 3000, 4500, and 8500 × g the oil recovery gradually increased from 6.52 to 8.26 to 9.72% (*w*/*w*), respectively, and stepwise increasing centrifugation time from 2 to 10 and to 20 min increased oil recovery from 4.87 to 6.55 to 8.26% (*w*/*w*), respectively (see supporting information Table 3). The recovered oil yield was similar, or in some cases, higher to that reported from herring co-products processed either by alkaline pH-shift processing or conventional heat-based separation; 0.5 and 6% (*w*/*w*), respectively (Abdollahi & Undeland, 2020). The gain in oil recovery with increasing g-force from 4500 to 8500 × g was very small, from 8.26 to 9.72%; thus, considering industrial applicability, it was decided to continue further with 4500 × g for 20 min.

**Fig. 4:**
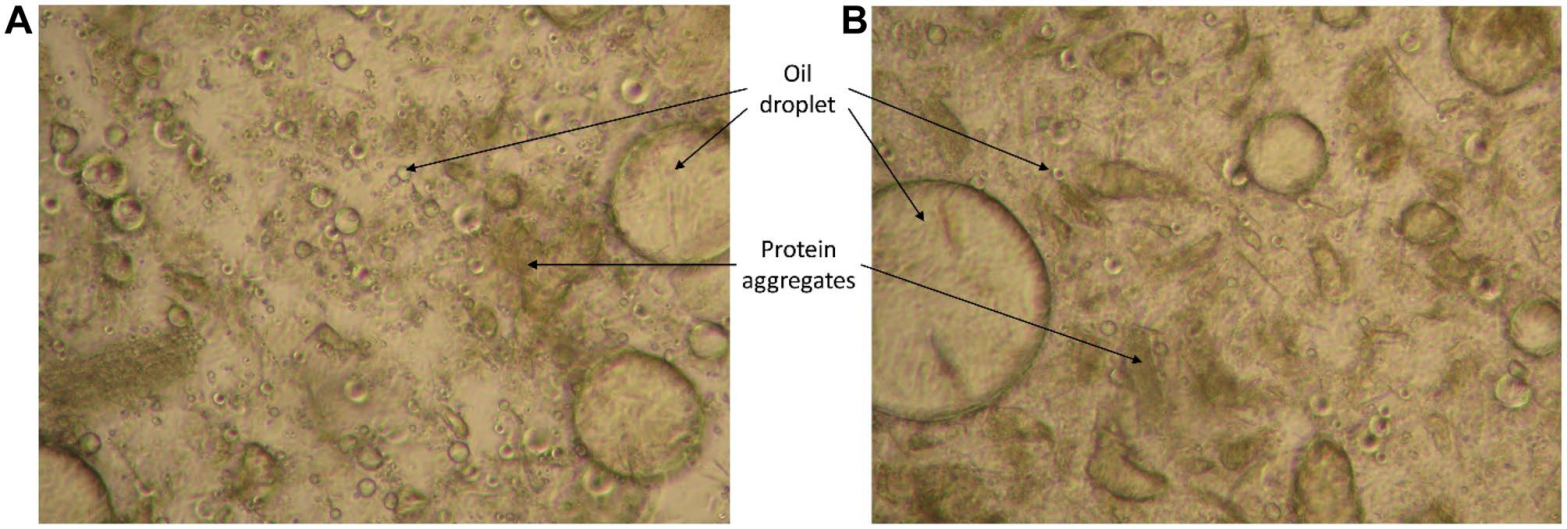
Emulsion formed at the oil-hydrolysate interface after centrifugation at 4500 × g for 20 min. **A** Non-heat-treated silage; **B** heat-treated silage. Images were taken with × 40 magnification as described earlier

Microscopic images of the emulsion phase formed between the oil and hydrolysates phases after centrifugation at 4500 × g for 20 min are shown in Fig. 4. In general, fewer oil droplets were noticed in the emulsion phase from the heat-treated silage, compared to the non-heat-treated one, which could possibly be due to heat-induced destabilization of the emulsions caused, e.g., by protein aggregation. The latter resulted in a coarse and non-homogeneous emulsion encompassing less oil with larger droplets. These large oil droplets could either be separated oil redispersed in the emulsion and/or droplets formed by coalescence. The latter is a phenomenon continuously happening in all emulsions at different rates, which destabilizes emulsions and causes phase separation (Krause, 1996; McClements, 2004). As a result, there was a higher oil recovery from the heat-treated silage (see Fig. 3A and supporting information Table 3).

The proximate composition of the herring co-product raw material, silage, and phases derived thereof after centrifugation at 4500 × g for 20 min are presented in Table 1. The proximate composition of the herring co-products used in our study was in line with the values reported by Aidos et al. (2002a, b), suggesting similarities in composition when herring raw materials are from the same catching season (i.e., September vs October). The small dilution of the raw material by acid and antioxidants at the start of the ensilaging did not create a significantly (*p* > 0.05) different composition of the produced silage. There was also no significant (*p* > 0.05) difference in protein contents between the silage and the hydrolysates derived thereof. However, significantly (*p* < 0.05) higher protein contents, compared to the silage and hydrolysates, were noticed in the solids. The hydrolysates contained similar ash contents to that of the co-products, silage, and solids, suggesting that some of the ash remains soluble in the hydrolysate phase. The oil phase contained 90–92% crude lipids, and there was no significant (*p* > 0.05) difference between oils from heat-treated and non-heat-treated silage.

**Table 1:** Proximate composition of herring co-products, silage, and phases derived thereof when subjecting non-heat-treated/heat-treated silage (85 °C; 30 min) to centrifugation at 4500 × g for 20 min. Results are expressed as mean ± SEM (*n* = 3). Different lower-case superscript letters within the same column denote significance (*p* < 0.05) differences

**Sample type**	**Treatment**	**Moisture (%)**	**Crude protein (%)**	**Crude lipid (%)**	**Ash (%)**
**Co-products**	n.a	73.26 ± 0.33^a^	11.12 ± 0.41^a^	11.26 ± 1.51^a^	2.36 ± 0.12^a^
**Silage; day: 2**	n.a	72.27 ± 0.04^b^	11.42 ± 0.02^a^	13.46 ± 0.29^a^	2.32 ± 0.02^a,e^
**Oil**	Non-heat	0.00^c^	0.29 ± 0.04^b^	90.33 ± 0.22^b^	0.00^b^
	Heat	0.16 ± 0.28^c^	0.19 ± 0.03^b^	92.12 ± 1.37^b^	0.00^b^
**Emulsion**	Non-heat	32.04 ± 0.02^d^	8.43 ± 0.05^c^	60.67 ± 0.56^c^	1.12 ± 0.02^c^
	Heat	62.48 ± 0.10^e^	13.87 ± 0.02^d^	24.20 ± 0.22^d^	1.86 ± 0.07^d,e^
**Hydrolysates**	Non-heat	84.98 ± 0.04^f^	10.93 ± 0.22^a^	0.15 ± 0.08^e^	2.46 ± 0.20^a^
	Heat	84.43 ± 0.03^f^	11.06 ± 0.09^a^	0.60 ± 0.15^e^	2.54 ± 0.12^a^
**Solids**	Non-heat	75.59 ± 0.29^ g^	15.17 ± 0.02^e^	6.62 ± 0.04^f^	2.56 ± 0.15^a^
	Heat	75.48 ± 0.03^ g^	16.67 ± 0.01^f^	4.26 ± 0.17^f^	2.58 ± 0.02^a^

*n.a.* not applicable

Oils from heat-treated and non-heat-treated silage had similar lightness (L* = 46–50), redness (a* = 0.36–2.62), and yellowness (b* = 30–34) values. However, oil from heat-treated silage had slightly, but, significantly (*p* < 0.05), lower lightness and higher yellowness values, compared to that from non-heat-treated silage; i.e.,L* values of 46.55 vs. 50.32, and b* values 34.33 vs. 30.12, respectively. The higher yellowness after heating could possibly be due to the formation of non-enzymatic browning reaction products (Koh et al., 2015), e.g., pyrroles (Zamora et al., 2007), something which requires further investigation.

The fatty acid composition of herring co-products, silage, and recovered fish oils is presented in Table 2. The oils contained 8% EPA and 11% DHA based on total fatty acids. This EPA content was in line with that previously reported by Isabel Aidos et al. (2002a, b), 7%, when recovering crude oil from herring co-products caught in May using heating and decanting. On the other hand, our analyzed DHA content was slightly higher than that reported in the mentioned study, 6%, possibly due to seasonal differences (Aidos et al., 2002a, b). There were no significant (*p* > 0.05) differences in total PUFA, LC n-3 PUFA, or LC n-6 PUFA contents between on the one hand co-products and silage, and on the other, between oils derived from heat-treated or native silage. The high content of LC MUFA (e.g., C20:1 n-9, gondoic acid and C22:1 n-11, cetoleic acid) recorded in this study is typical for oils from herring and mackerel (Yang et al., 2016), and are further to the LC n-3 PUFA themselves, interesting as a few animal studies have indicated they may prevent life-style related diseases, such as type 2 diabetes, metabolic syndrome, and atherosclerosis. For example, mice-studies have revealed that the mentioned LC MUFA can improve endothelial function by altering the microbial flora (Tsutsumi et al., 2021) as well as the glucose/lipid homeostasis (Yang et al., 2011). Cetoleic acid may also improve the efficiency of the conversion of ALA to EPA and DHA (Østbye et al., 2019).

**Table 2:** Fatty acid composition (mg FAME/g) of herring co-product, silage, and fish oil. Fish oils were recovered by subjecting non-heat-treated/ heat-treated silage to centrifugation at 4500 × g for 20 min. Results are expressed as mean ± SEM (*n* = 3). Different lower-case superscript letters within the same row denote significance (*p* < 0.05) differences. ^1^LC: Chain length > C18, PUFA > 1 double bond starting in n-3 or n-6 position

**FAME**	**Co-product**	**Silage; 2 d**	**Oils recovered from**	
			**Non-heat-treated silage**	**Heat-treated silage**
C10:0	0.00 ± 0.00	0.00 ± 0.00	0.02 ± 0.00	0.02 ± 0.00
C12:0	0.02 ± 0.00	0.02 ± 0.00	0.16 ± 0.00	0.16 ± 0.00
C12:1	0.00 ± 0.00	0.00 ± 0.00	0.02 ± 0.00	0.02 ± 0.00
C14:0	1.80 ± 0.16	1.69 ± 0.09	13.88 ± 0.51	14.87 ± 0.42
C14:1	0.01 ± 0.00	0.01 ± 0.00	0.12 ± 0.00	0.13 ± 0.01
C16:0	3.61 ± 0.28	3.58 ± 0.17	28.62 ± 1.01	30.96 ± 0.83
C16:1	1.57 ± 0.13	1.49 ± 0.09	12.37 ± 0.46	13.04 ± 0.38
C18:0	0.35 ± 0.02	0.36 ± 0.02	2.79 ± 0.10	3.03 ± 0.11
C18:1 (oleate)	2.18 ± 0.15	3.04 ± 0.15	24.90 ± 0.93	26.23 ± 0.79
C18:1 (vaccenate)	1.88 ± 0.13	2.62 ± 0.13	21.52 ± 0.80	22.67 ± 0.69
C18:2	0.44 ± 0.03	0.54 ± 0.03	4.47 ± 0.19	4.61 ± 0.16
C18:3	0.39 ± 0.03	0.37 ± 0.02	3.05 ± 0.10	3.20 ± 0.13
C20:0	0.03 ± 0.00	0.04 ± 0.00	0.24 ± 0.03	0.34 ± 0.02
C20:1	2.03 ± 0.19	1.88 ± 0.09	15.51 ± 0.54	16.14 ± 0.59
C20:2 n6	0.06 ± 0.00	0.06 ± 0.00	0.48 ± 0.01	0.50 ± 0.02
C20:4 n6	0.09 ± 0.01	0.09 ± 0.01	0.75 ± 0.02	0.75 ± 0.04
C20:3 n3	0.04 ± 0.00	0.04 ± 0.00	0.31 ± 0.02	0.32 ± 0.01
**C20:5 n3 (EPA)**	**1.83 ± **0.12^a^	**1.85 ± **0.07^a^	**15.15 ± **0.59^b^	**15.91 ± **0.51^b^
C22:1	3.93 ± 0.37	3.63 ± 0.16	30.01 ± 1.22	31.58 ± 1.01
C22:5 n3	0.14 ± 0.02	0.13 ± 0.02	1.17 ± 0.03	1.25 ± 0.05
**C22:6 n3 (DHA)**	**2.68 ± **0.19^a^	**2.78 ± **0.11^a^	**22.90 ± **0.93^b^	**23.92 ± **0.79^b^
C24:1	0.16 ± 0.00	0.15 ± 0.01	1.16 ± 0.12	1.21 ± 0.11
**Total SFA**	**5.80 ± 0.46**^**a**^	**5.69 ± 0.29**^**a**^	**45.71 ± 1.63**^**b**^	**49.37 ± 1.38**^**b**^
**Total MUFA**	**7.70 ± 0.70**^**a**^	**7.16 ± 0.34**^**a**^	**59.18 ± 2.10**^**b**^	**62.12 ± 2.09**^**b**^
**Total PUFA**	**5.66 ± 0.41**^**a**^	**5.87 ± 0.26**^**a**^	**48.28 ± 1.80**^**b**^	**50.47 ± 1.61**^**b**^
**Sum LC n-3 PUFA**^1^	**4.69 ± 0.33**^**a**^	**4.81 ± 0.20**^**a**^	**39.53 ± 1.50**^**b**^	**41.41 ± 1.27**^**b**^
**Sum LC n-6 PUFA**^1^	**0.15 ± 0.02**^**a**^	**0.15 ± 0.01**^**a**^	**1.23 ± 0.01**^**b**^	**1.26 ± 0.06**^**b**^

**Fig. 5:**
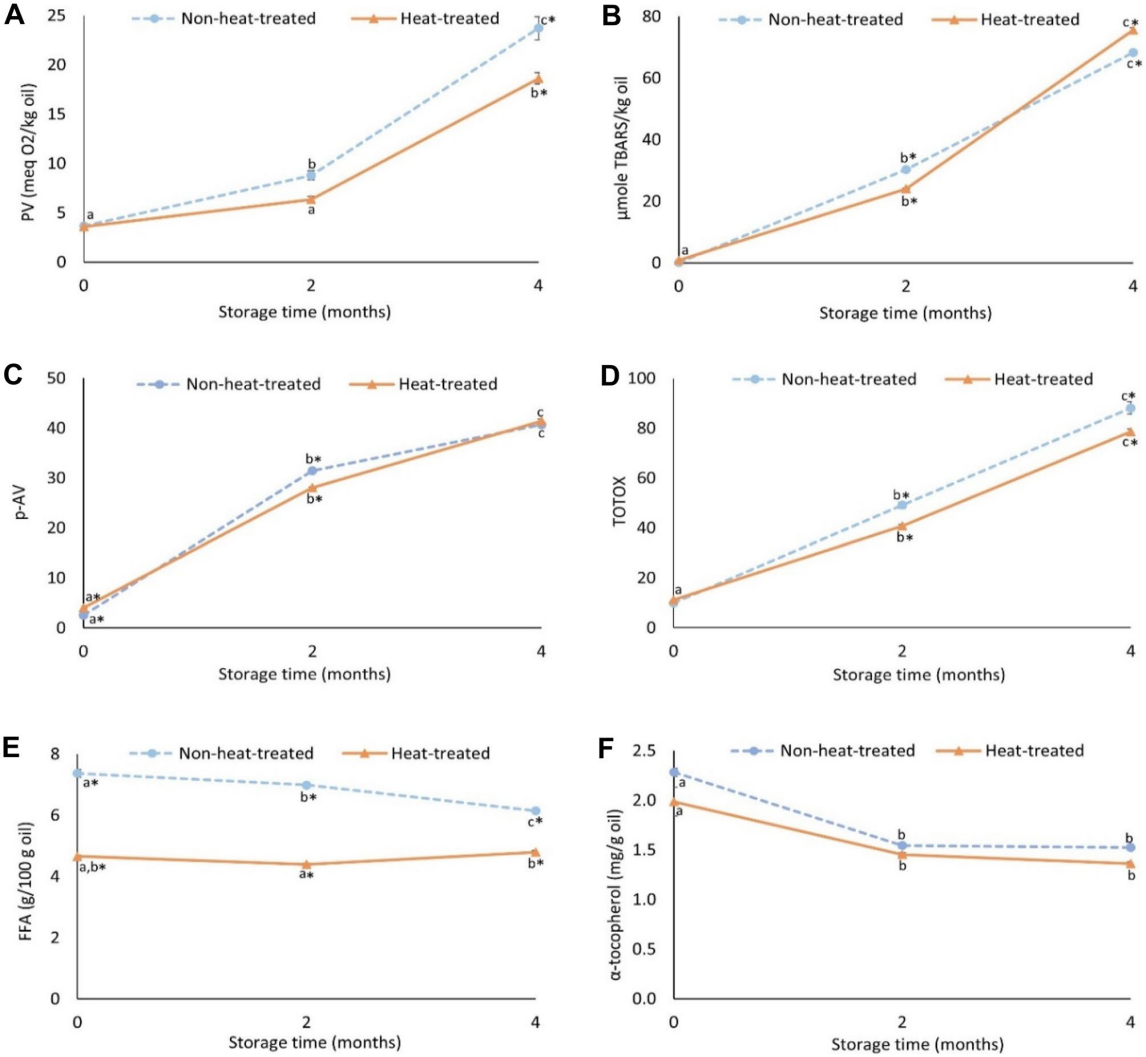
Changes in PV (**A**), TBARS (**B**), p-AV (**C**), TOTOX (**D**), FFA (**E**), and α-tocopherol (**F**) values during storage of oils from non-heat-treated and heat-treated silages at 4 °C for 0–4 months. Results are expressed as mean ± SEM (*n* = 3). Different lower-case letters along the same line denote significance (*p* < 0.05) difference; star (*) sign represents significant (*p* < 0.05) difference between treatments at different time points

To investigate the oxidative stability, oils were stored at 4 °C for 0–4 months, and changes in PV, TBARS, p-AV, TOTOX, FFA, and α-tocopherol were monitored (Fig. 5). At day 0, the recovered oils had PV, p-AV, and TOTOX values in the range of 3.6–3.7 meq/kg oil, 2.5–4.0, and 9.9–11.1, respectively, which were within acceptable limits of 5 meq/kg (PV), 20 (p-AV), and 26 (TOTOX), respectively, for human consumption specified by the GOED voluntary monograph (GOED, 2021; Ismail et al., 2016). These values were also in agreement with oil recovered from garfish (*Belone belone*, L. 1758), golden mullet (*Mugil auratus*, Risso 1810), and shad (*Alosa fallax*, Lacepede 1803) using solvent extraction (Boran et al., 2006). However, during storage at 4 °C, PV, p-AV, and TOTOX, as well as the initial TBARS values, increased significantly (*p* < 0.05). Similar observations have also been reported for many other fish oils, as reported, e.g., by Boran et al. (2006). In our study, significantly (*p* < 0.05) lower PVs were recorded in oil from heat-treated silage, compared to in oils from the non-heat-treated silage, at the end of the 4-month storage. For TBARS and p-AV, there were no such differences. The fact that peroxides accumulated less in oils from silage subjected to heat could be due to different oxidation kinetics caused, e.g., by the lower FFA content in this oil (Chew, 2020). Also, it could be due to the inactivation of catalytic enzymes such as lipoxygenases (LOX) during heating of the silage (Sajib et al., 2022). Wu et al. (2022) recently reported high LOX-activity in herring heads, which stimulated severe oxidation during their cold storage.

The endogenous antioxidant α-tocopherol was significantly (*p* < 0.05) consumed during storage, supporting the enhanced oxidation. Oil from heat-treated silage had significantly (*p* < 0.05) lower FFA values already from the start of the storage, compared to the non-heat-treated one, which could possibly be due to a reduction in lipase activities (Dalheim et al., 2021; Li et al., 2016; Wang et al., 1991). The FFA content then only decreased significantly (*p* < 0.05) in the oil from non-heat-treated silage, most likely due to oxidation of this lipid class. Although the oil from heat-treated silage only had FFA values of 4–5%, which is within the quality guidelines of 2–5% for crude fish oil followed by industries (Oterhals & Vogt, 2013), further refining of this oil might be needed to remove e.g. peroxides and compounds yielding off-flavor, before being used in food applications (Oterhals & Vogt 2013).

**Fig. 6:**
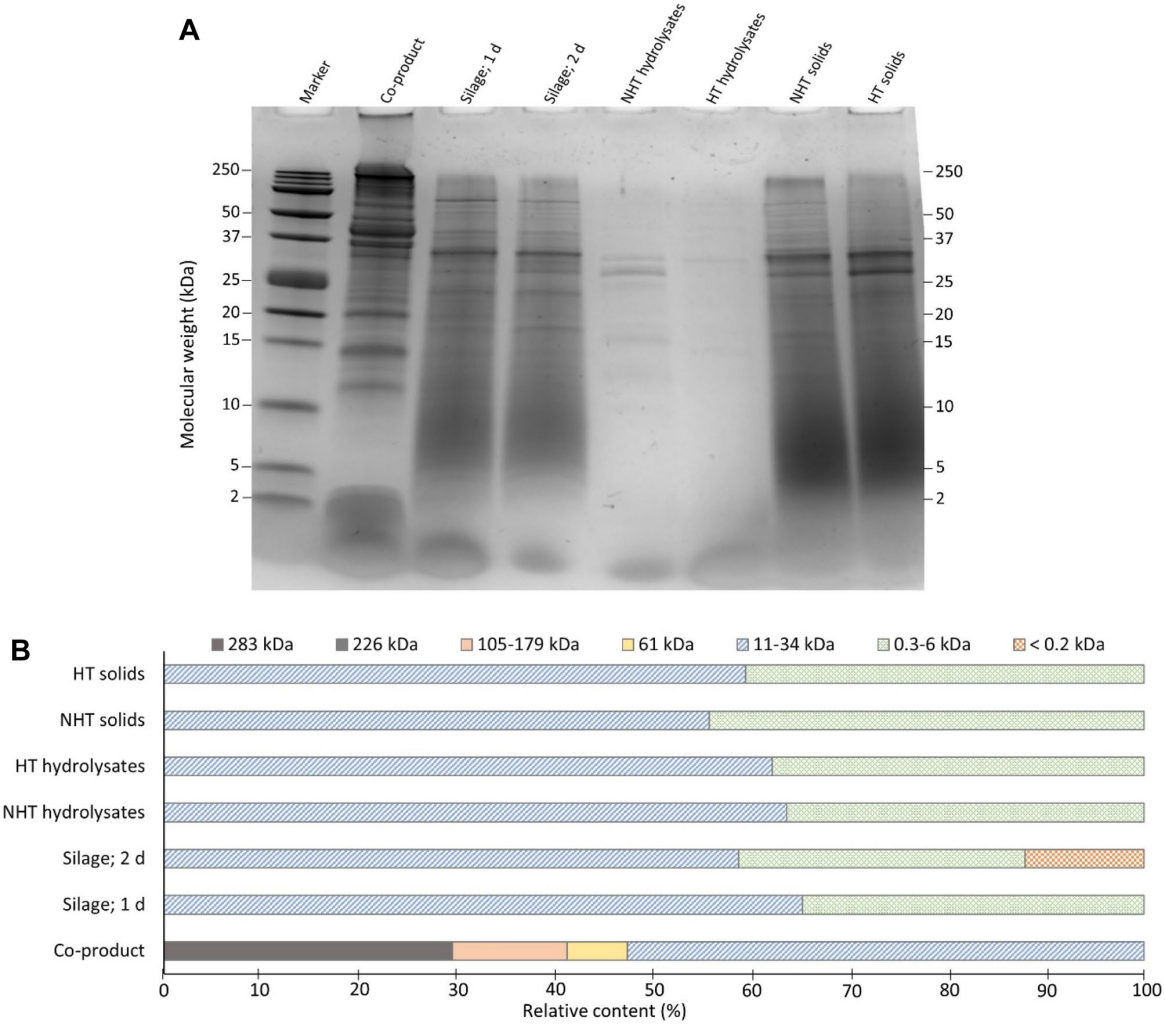
Relative content of differently sized proteins and peptides in co-product, silages, protein hydrolysates, and solids recovered after centrifugation at 4500 × g for 20 min. Molecular weight distribution analyzed by SDS-PAGE (**A**) and HP-SEC (**B**). NHT: non-heat-treated silage; HT: heat-treated silage

The relative content of differently sized proteins and peptides in co-product, silage, protein hydrolysates, and solids as measured by SDS-PAGE and HP-SEC is shown in Fig. 6. Results confirmed that high molecular weight (HMW) proteins (100 to ≥ 250 kDa) in co-products were hydrolyzed into smaller peptides after 1–2 days of ensilaging. As a result, dense bands around 11–39 kDa and 2–10 kDa were noticed in the silage (Fig. 6A). The solids contained both small peptides 2–10 kDa and HMW proteins in the molecular weight range of 28–250 kDa; the latter was slightly more enriched in solids recovered from heat-treated silage, possibly due to protein-crosslinking upon heat treatment (Xiong et al., 2021; Xu et al., 2020). Short-chain peptides and free amino acids < 2 kDa were not detected by the SDS-PAGE analysis as they escape the gel, something which can explain why there were barely any bands in lanes representing the hydrolysate fractions. Therefore, HP-SEC was used to deepen the understanding of the MW distribution of soluble proteins and peptides (Fig. 6B). In agreement with the SDS-PAGE image, the relative content of HMW proteins ~ 283 kDa in the co-products decreased after 1–2 days of ensilaging, resulting in short-chain peptides of 0.3–6 kDa. Also in our earlier lab-scale study, silage rich in short-chain peptides of 1.9–4.7 kDa was obtained after 3 days of ensilaging of herring co-product (Sajib et al., 2020). In the hydrolysates recovered from pre-heated or native silages, there was no relative difference in the content of short-chain peptides of 0.3–6 kDa. That the solids also contained short-chain peptides reflects that their moisture component (~ 75%) has a composition that mirrors that of the soluble hydrolysate fraction. It is also possible that some peptides precipitate during centrifugation. A possible route to produce more short-chain peptides < 1 kDa and thereby further raise the bioactivity of silage-derived hydrolysates (Liu et al., 2016; Zou et al., 2016) would be to add exogenous endopeptidases before or after separation of the hydrolysate.

The amino acid composition of herring co-products and silage, as well as protein hydrolysates and solids obtained after centrifugation at 4500 × g for 20 min, is presented in Table 3. There was no significant (*p* > 0.05) difference in total amino acid contents between co-product and silage, suggesting that co-product nutrients were well-preserved during ensilaging and the small dilution by acid and antioxidant did not affect the nutrient density. The silage and non-oil products derived thereof contained all essential amino acids except tryptophan, which is not recovered by the method used here. Among essential amino acids, lysine was the most abundant in all samples, followed by leucine, valine, threonine, isoleucine, phenylalanine, methionine, and histidine. The fact that solids contained significantly (*p* < 0.05) higher levels of total amino acids, compared to the hydrolysates, agrees with the crude protein data and suggests that these solids can also be used as a separate product after the recovery of oil and hydrolysates.

**Table 3:** Amino acid composition (mg amino acid/g wet sample) of herring co-product, silage, and phases derived thereof after subjecting non-heat-treated/heat-treated silage to centrifugation at 4500 × g for 20 min. Results are expressed mean ± SEM; *n* = 3. Different lower-case superscript letters within the same row denote significance (*p* < 0.05) differences

**Amino acid**	**Co-product**	**Silage; day 2**	**Hydrolysates recovered from**		**Solids recovered from**	
			**Non-heat-treated silage**	**Heat-treated silage**	**Non-heat-treated silage**	**Heat-treated silage**
Glycine	15.87 ± 1.01	18.40 ± 0.22	18.99 ± 0.67	21.00 ± 0.45	26.33 ± 1.05	25.64 ± 0.23
Alanine	18.19 ± 0.35	18.82 ± 0.07	18.86 ± 0.37	19.55 ± 0.16	24.92 ± 0.04	26.68 ± 0.57
Serine	12.61 ± 0.12	12.69 ± 0.16	12.51 ± 0.30	12.55 ± 0.26	16.70 ± 0.08	17.63 ± 0.55
Proline	11.57 ± 0.43	12.86 ± 0.09	14.10 ± 0.16	14.24 ± 0.08	17.55 ± 0.23	17.64 ± 0.32
Valine	15.78 ± 0.36	15.50 ± 0.16	15.69 ± 0.23	15.64 ± 0.15	21.53 ± 0.21	24.50 ± 0.54
Threonine	13.63 ± 0.14	13.18 ± 0.33	12.16 ± 0.29	12.23 ± 0.08	17.04 ± 0.13	18.58 ± 0.35
Isoleucine	11.77 ± 0.38	11.59 ± 0.07	10.66 ± 0.24	10.58 ± 0.14	16.53 ± 0.47	19.12 ± 0.48
Leucine	22.36 ± 0.55	21.38 ± 0.28	21.90 ± 0.40	21.87 ± 0.10	30.86 ± 0.27	35.39 ± 0.48
Aspartic acid	28.18 ± 0.50	28.09 ± 0.23	23.95 ± 0.47	23.74 ± 0.23	34.28 ± 0.30	37.22 ± 0.17
Lysine	26.56 ± 0.45	25.01 ± 0.06	26.14 ± 0.21	25.31 ± 0.46	32.97 ± 0.49	36.27 ± 0.49
Glutamic acid	39.91 ± 0.77	39.10 ± 0.44	35.55 ± 0.52	34.18 ± 0.22	49.59 ± 0.63	55.41 ± 1.26
Methionine	8.31 ± 0.06	7.82 ± 0.04	8.08 ± 0.10	8.30 ± 0.09	12.00 ± 0.03	13.63 ± 0.29
Histidine	7.43 ± 0.06	7.35 ± 0.14	6.86 ± 0.07	6.73 ± 0.05	8.51 ± 0.06	9.25 ± 0.27
Phenylalanine	11.52 ± 0.17	11.15 ± 0.04	10.56 ± 0.20	10.51 ± 0.03	17.16 ± 0.20	19.58 ± 0.34
Arginine	15.37 ± 0.31	16.36 ± 0.77	14.82 ± 0.20	14.78 ± 0.12	19.14 ± 0.53	19.97 ± 0.48
Tyrosine	8.96 ± 0.18	8.68 ± 0.17	6.65 ± 0.23	6.65 ± 0.24	10.67 ± 0.23	12.23 ± 0.48
**TAA**	**268.03** ± 1.66^a^	**267.98** ± 1.55^a^	**257.46** ± 3.80^a^	**257.85** ± 1.24^a^	**355.76** ± 1.01^b^	**388.73** ± 6.49^c^
**TEAA**	**117.36** ± 1.82^a^	**112.96** ± 0.37^a^	**112.04** ± 1.40^a^	**111.16** ± 0.90^a^	**156.60** ± 1.22^b^	**176.31** ± 2.97^c^
**TEAA/TAA**	**0.44**	**0.42**	**0.44**	**0.43**	**0.44**	**0.45**

*TAA* total amino acids, *TEAA* total essential amino acids

## Conclusion

This study showed that DH and lipid oxidation of herring co-product silages produced in pilot-scale agreed well with earlier lab-scale data (e.g. 38% DH vs. 40% DH after 2 days). Batch centrifugation of the produced silage at 3000–8500 × g for 2–20 min allowed recovery of fish oil and protein hydrolysates, with centrifugation at 8500 × g for 20 min resulting in the highest recovery of oil (9.7% *w*/*w*) and hydrolysates (53.0% *w*/*w*). Heat-treating the silage (85 °C; 30 min) prior to centrifugation resulted in a higher oil and hydrolysates recovery, compared to the non-heat-treated silage. The recovered oil contained 8 and 11% EPA and DHA, respectively, of total fatty acids. In addition, there were significant levels of the interesting LC MUFA gondoic acid and cetoleic acid. Also, the oils had PV, p-AV, and TOTOX values in the range of 3.6–3.7 meq/kg oil, 2.5–4.0, and 9.9–11.1, respectively, which were within acceptable limits of 5 meq/kg (PV), 20 (p-AV), and 26 (TOTOX), respectively, for human consumption specified by the GOED voluntary monograph (GOED, 2021). However, these values increased during storage at 4 °C for 0–4 months, stressing the importance of appropriate storage conditions, e.g., cold temperature, darkness, excluding oxygen etc. and/or addition of extra antioxidants into the oil. The recovered protein hydrolysates contained around 37% short-chain peptides in the MW range 0.3–6 kDa and around 63% peptides in the MW range 11–34 kDa. The silage, hydrolysates, and solids contained all measured essential amino acids, and therefore would be good peptide/protein-sources for both food and feed applications. The results of this study thus suggest that herring co-product silage can be a valuable source of both fish oil and protein hydrolysates, paving the way for ensilaging-based biorefining of herring side streams into multiple products.

## Supplementary Information

Below is the link to the electronic supplementary material.

Supplementary file1 (DOCX 630 KB)

## Data Availability

The datasets supporting the findings of this article are available upon reasonable request.

## References

[CR1] Abdollahi M, Axelsson J, Carlsson N-G, Nylund GM, Albers E, Undeland I (2019). Effect of stabilization method and freeze/thaw-aided precipitation on structural and functional properties of proteins recovered from brown seaweed (Saccharina latissima). Food Hydrocolloids.

[CR2] Abdollahi, M., Marmon, S., Chaijan, M., & Undeland, I. (2016). Tuning the pH-shift protein-isolation method for maximum hemoglobin-removal from blood rich fish muscle. *Food Chemistry, 212*(Supplement C), 213–224. http://www.sciencedirect.com/science/article/pii/S030881461630855X 10.1016/j.foodchem.2016.05.165 27374526

[CR3] Abdollahi, M., & Undeland, I. (2020). A novel cold biorefinery approach for isolation of high quality fish oil in parallel with gel-forming proteins. *Food Chemistry*, 127294. 10.1016/j.foodchem.2020.127294 32615378

[CR4] Aidos I, Lourenclo S, Van der Padt A, Luten J, Boom R (2002). Stability of crude herring oil produced from fresh byproducts: Influence of temperature during storage. Journal of Food Science.

[CR5] Aidos I, van der Padt A, Luten JB, Boom RM (2002). Seasonal changes in crude and lipid composition of herring fillets, byproducts, and respective produced oils. Journal of Agricultural and Food Chemistry.

[CR6] Arason, S., Thoroddsson, G., & Valdimarsson, G. (1990). The production of silage from waste and industrial fish: The Icelandic experience. *Marketing profit out of seafood wastes. Proceeding of the International Conference on Fish By-products* (pp. 79–85).

[CR7] Boran G, Karaçam H, Boran M (2006). Changes in the quality of fish oils due to storage temperature and time. Food Chemistry.

[CR8] Chew SC, Ramadan MF (2020). Chapter 7 - Cold pressed rapeseed (Brassica napus) oil. Cold Pressed Oils.

[CR9] Dalheim L, Svenning JB, Eilertsen HC, Vasskog T, Olsen RL (2021). Stability of lipids during wet storage of the marine diatom Porosira glacialis under semi-preserved conditions at 4 and 20 C. Journal of Applied Phycology.

[CR10] Darwesh, A. M., Bassiouni, W., Sosnowski, D. K., & Seubert, J. M. (2020). Can N-3 polyunsaturated fatty acids be considered a potential adjuvant therapy for COVID-19-associated cardiovascular complications? *Pharmacology & Therapeutics*, 107703. 10.1016/j.pharmthera.2020.107703 PMC7534795 33031856

[CR11] de la Fuente B, Tornos A, Príncep A, Lorenzo JM, Pateiro M, Berrada H, Martí-Quijal FJ, Lorenzo JM, Barba FJ (2020). Chapter Six - scaling-up processes: Patents and commercial applications. Advances in Food and Nutrition Research.

[CR12] Endo J, Arita M (2016). Cardioprotective mechanism of omega-3 polyunsaturated fatty acids. Journal of Cardiology.

[CR13] Ghaly, A. E., Ramakrishnan, V. V., Brooks, M. S., Budge, S. M., & Dave, D. (2013). Fish processing wastes as a potential source of proteins, amino acids and oils: A critical review. *Journal of Microbial & Biochemical Technology, 05*(04).

[CR14] GOED. (2021). The GOED voluntary monograph*.* Retrieved from: https://goedomega3.com/goed-monograph Accessed 2022.

[CR15] Harrysson, H., Hayes, M., Eimer, F., Carlsson, N.-G., Toth, G. B., & Undeland, I. (2018). Production of protein extracts from Swedish red, green, and brown seaweeds, Porphyra umbilicalis Kützing, Ulva lactuca Linnaeus, and Saccharina latissima (Linnaeus) JV Lamouroux using three different methods. *Journal of Applied Phycology*, 1–16.

[CR16] Hilmarsdottir GS, Ogmundarson Ó, Arason S, Gudjónsdóttir M (2020). The effects of varying heat treatments on lipid composition during pelagic fishmeal production. Processes.

[CR17] Hilmarsdottir GS, Ogmundarson Ó, Arason S, Gudjónsdóttir M (2021). Efficiency of fishmeal and fish oil processing of different pelagic fish species: Identification of processing steps for potential optimization toward protein production for human consumption. Journal of Food Processing and Preservation.

[CR18] Hilmarsdóttir, G. S., Ögmundarson, Ó., Arason, S., & Gudjónsdóttir, M. (2022). Identification of environmental hotspots in fishmeal and fish oil production towards the optimization of energy-related processes. *Journal of Cleaner Production, 343*, 130880. 10.1016/j.jclepro.2022.130880

[CR19] Hirafuji M, Machida T, Hamaue N, Minami M (2003). Cardiovascular protective effects of n-3 polyunsaturated fatty acids with special emphasis on docosahexaenoic acid. Journal of Pharmacological Sciences.

[CR20] Ismail A, Bannenberg G, Rice HB, Schutt E, MacKay D (2016). Oxidation in EPA-and DHA-rich oils: An overview. Lipid Technology.

[CR21] Jump DB, Depner CM, Tripathy S (2012). Omega-3 fatty acid supplementation and cardiovascular disease: Thematic review series: New lipid and lipoprotein targets for the treatment of cardiometabolic diseases. Journal of Lipid Research.

[CR22] Koh E, Ryu D, Surh J (2015). Ratio of malondialdehyde to hydroperoxides and color change as an index of thermal oxidation of linoleic acid and linolenic acid. Journal of Food Processing and Preservation.

[CR23] Krause, J. P. (1996). Emulsions and emulsion stability. *Food / Nahrung, 40*(6), 349–349. 10.1002/food.19960400617

[CR24] Larsson KJ, Undeland IK (2010). Effect of caffeic acid on haemoglobin-mediated lipid and protein oxidation in washed cod mince during ice and frozen storage. Journal of the Science of Food and Agriculture.

[CR25] Lee CM, Trevino B, Chaiyawat M (1996). A simple and rapid solvent extraction method for determining total lipids in fish tissue. Journal of AOAC International.

[CR26] Li B, Zhao L, Chen H, Sun D, Deng B, Li J, Wang F (2016). Inactivation of lipase and lipoxygenase of wheat germ with temperature-controlled short wave infrared radiation and its effect on storage stability and quality of wheat germ oil. PLoS ONE.

[CR27] Liaset, B., Nortvedt, R., Lied, E., & Espe, M. (2002). Studies on the nitrogen recovery in enzymic hydrolysis of Atlantic salmon (Salmo salar, L.) frames by Protamex™ protease. *Process Biochemistry, 37*(11), 1263–1269.

[CR28] Liu R, Xing L, Fu Q, Zhou G-H, Zhang W-G (2016). A review of antioxidant peptides derived from meat muscle and by-products. Antioxidants.

[CR29] Liu, Y., Ramakrishnan, V. V., & Dave, D. (2020a). Enzymatic hydrolysis of farmed Atlantic salmon by-products: Investigation of operational parameters on extracted oil yield and quality. *Process Biochemistry*.

[CR30] Liu Y, Ramakrishnan VV, Dave D (2020). Lipid class and fatty acid composition of oil extracted from Atlantic salmon by-products under different optimization parameters of enzymatic hydrolysis. Biocatalysis and Agricultural Biotechnology.

[CR31] Lowry RR, Tinsley IJ (1976). Rapid colorimetric determination of free fatty acids. Journal of the American Oil Chemists' Society.

[CR32] Mariotti, F., Tomé, D., & Mirand, P. P. (2008). Converting nitrogen into protein—Beyond 6.25 and Jones' factors. *Critical reviews in food science and nutrition, 48*(2), 177–184. 10.1080/10408390701279749 18274971

[CR33] Markets, R. a. (2021). Fish protein hydrolysate market forecast to 2027 - COVID-19 impact and global analysis by technology; form; source and application, and geography*.* Retrieved from: https://www.researchandmarkets.com/reports/5006424/fish-protein-hydrolysate-market-forecast-to-2027 Accessed 2021–06–07 2021.

[CR34] Markwell MAK, Haas SM, Bieber L, Tolbert N (1978). A modification of the Lowry procedure to simplify protein determination in membrane and lipoprotein samples. Analytical Biochemistry.

[CR35] Marseno DW, Indrati R, Ohta Y (1998). A simplified method for determination of free fatty acids for soluble and immobilized lipase assay. Indonesian Food and Nutrition Progress.

[CR36] Mbatia B, Adlercreutz D, Adlercreutz P, Mahadhy A, Mulaa F, Mattiasson B (2010). Enzymatic oil extraction and positional analysis of ω-3 fatty acids in Nile perch and salmon heads. Process Biochemistry.

[CR37] McClements, D. J. (2004). *Food emulsions: Principles, practices, and techniques*: CRC press.

[CR38] Meisel H, FitzGerald RJ (2003). Biofunctional peptides from milk proteins: Mineral binding and cytomodulatory effects. Current Pharmaceutical Design.

[CR39] Melgosa R, Marques M, Paiva A, Bernardo A, Fernández N, Sá-Nogueira I, Simões P (2021). Subcritical water extraction and hydrolysis of cod (Gadus morhua) frames to produce bioactive protein extracts. Foods.

[CR40] Nielsen P, Petersen D, Dambmann C (2001). Improved method for determining food protein degree of hydrolysis. Journal of Food Science.

[CR41] Nikoo M, Benjakul S, Rahmanifarah K (2016). Hydrolysates from marine sources as cryoprotective substances in seafoods and seafood products. Trends in Food Science and Technology.

[CR42] Oliver L, Dietrich T, Marañón I, Villarán MC, Barrio RJ (2020). Producing omega-3 polyunsaturated fatty acids: A review of sustainable sources and future trends for the EPA and DHA market. Resources.

[CR43] Olsen RL, Toppe J (2017). Fish silage hydrolysates: Not only a feed nutrient, but also a useful feed additive. Trends in Food Science & Technology.

[CR44] Østbye T-KK, Berge GM, Nilsson A, Romarheim OH, Bou M, Ruyter B (2019). The long-chain monounsaturated cetoleic acid improves the efficiency of the n-3 fatty acid metabolic pathway in Atlantic salmon and human HepG2 cells. British Journal of Nutrition.

[CR45] Oterhals, Å., & Vogt, G. (2013). Impact of extraction, refining and concentration stages on the stability of fish oil. In *Food Enrichment with Omega-3 Fatty Acids* (pp. 111–129): Elsevier.

[CR46] Özyurt G, Özkütük AS, Uçar Y, Durmuş M, Özoğul Y (2018). Fatty acid composition and oxidative stability of oils recovered from acid silage and bacterial fermentation of fish (Sea bass–Dicentrarchus labrax) by-products. International Journal of Food Science & Technology.

[CR47] Rodrigues, D. P., Calado, R., Ameixa, O. M., Valcarcel, J., & Vázquez, J. A. (2021). Valorisation of Atlantic codfish (Gadus morhua) frames from the cure-salting industry as fish protein hydrolysates with in vitro bioactive properties. *LWT - Food Science and Technology*, 111840.

[CR48] Sajib, M., Albers, E., Langeland, M., & Undeland, I. (2020). Understanding the effect of temperature and time on protein degree of hydrolysis and lipid oxidation during ensilaging of herring (Clupea harengus) filleting co-products. *Scientific reports, 10*(1), 9590. 10.1038/s41598-020-66152-0 10.1038/s41598-020-66152-0 PMC7293326 32533006

[CR49] Sajib M, Langeland M, Undeland I (2022). Effect of antioxidants on lipid oxidation in herring (Clupea harengus) co-product silage during its production, heat-treatment and storage. Scientific Reports.

[CR50] Sajib, M., & Undeland, I. (2020). Towards valorization of herring filleting by-products to silage 2.0: Effect of temperature and time on lipid oxidation and non-enzymatic browning reactions. *LWT - Food Science and Technology*, 109441. http://www.sciencedirect.com/science/article/pii/S0023643820304308

[CR51] Santos, C. E. d., Silva, J. d., Zinani, F., Wander, P., & Gomes, L. P. (2015). Oil from the acid silage of Nile tilapia waste: Physicochemical characteristics for its application as biofuel. *Renewable Energy,**80*, 331–337. 10.1016/j.renene.2015.02.028

[CR52] Sarmadi, B. H., & Ismail, A. (2010). Antioxidative peptides from food proteins: A review. *Peptides, 31*(10), 1949–1956. http://www.sciencedirect.com/science/article/pii/S0196978110002640 10.1016/j.peptides.2010.06.020 20600423

[CR53] Schmedes A, Hølmer G (1989). A new thiobarbituric acid (TBA) method for determining free malondialdehyde (MDA) and hydroperoxides selectively as a measure of lipid peroxidation. Journal of the American Oil Chemists Society.

[CR54] Semb, T. N. (2012). Analytical methods for determination of the oxidative status in oils. Institutt for bioteknologi.

[CR55] Šližyte R, Daukšas E, Falch E, Storrø I, Rustad T (2005). Yield and composition of different fractions obtained after enzymatic hydrolysis of cod (Gadus morhua) by-products. Process Biochemistry.

[CR56] Šližytė, R., Opheim, M., Storrø, I., & Sterten, H. (2017). Simple technologies for converting rest raw materials of Atlantic salmon (Salmo salar) into high-quality, valuable, and tasty feed ingredients. *Journal of Aquatic Food Product Technology*, 1–16.

[CR57] Šližytė, R., Rustad, T., & Storrø, I. (2005). Enzymatic hydrolysis of cod (Gadus morhua) by-products: Optimization of yield and properties of lipid and protein fractions. *Process Biochemistry, 40*(12), 3680–3692. 10.1016/j.procbio.2005.04.007

[CR58] Sluiter, A., Hames, B., Hyman, D., Payne, C., Ruiz, R., Scarlata, C., . . . Wolfe, J. (2008). Determination of total solids in biomass and total dissolved solids in liquid process samples. *National Renewable Energy Laboratory, 9*.

[CR59] Sluiter, A., Hames, B., Ruiz, R., Scarlata, C., Sluiter, J., & Templeton, D. (2005). Determination of ash in biomass (NREL/TP-510–42622). *National Renewable Energy Laboratory, Golden*.

[CR60] Sun J, He H, Xie BJ (2004). Novel antioxidant peptides from fermented mushroom Ganoderma lucidum. Journal of Agricultural and Food Chemistry.

[CR61] Szabó Z, Marosvölgyi T, Szabó É, Bai P, Figler M, Verzár Z (2020). The potential beneficial effect of EPA and DHA supplementation managing cytokine storm in coronavirus disease. Frontiers in Physiology.

[CR62] Tsutsumi R, Yamasaki Y, Takeo J, Miyahara H, Sebe M, Bando M, Ueshima N (2021). Long-chain monounsaturated fatty acids improve endothelial function with altering microbial flora. Translational Research.

[CR63] Undeland I, Hultin HO, Richards MP (2002). Added triacylglycerols do not hasten hemoglobin-mediated lipid oxidation in washed minced cod muscle. Journal of Agricultural and Food Chemistry.

[CR64] van't Land, M., Vanderperren, E., & Raes, K. (2017). The effect of raw material combination on the nutritional composition and stability of four types of autolyzed fish silage. *Animal Feed Science and Technology*.

[CR65] Wang YJ, Miller LA, Addis PB (1991). Effect of heat inactivation of lipoxygenase on lipid oxidation in lake herring (Coregonus artedii). Journal of the American Oil Chemists' Society.

[CR66] Weill P, Plissonneau C, Legrand P, Rioux V, Thibault R (2020). May omega-3 fatty acid dietary supplementation help reduce severe complications in Covid-19 patients?. Biochimie.

[CR67] Wu, H., Abdollahi, M., & Undeland, I. (2021). Effect of recovery technique, antioxidant addition and compositional features on lipid oxidation in protein enriched products from cod-salmon and herring backbones. *Food Chemistry*, 129973. 10.1016/j.foodchem.2021.129973 33989878

[CR68] Wu H, Forghani B, Abdollahi M, Undeland I (2022). Lipid oxidation in sorted herring (Clupea harengus) filleting co-products from two seasons and its relationship to composition. Food Chemistry.

[CR69] Xiong, L., Boeren, S., Vervoort, J., & Hettinga, K. (2021). Effect of milk serum proteins on aggregation, bacteriostatic activity and digestion of lactoferrin after heat treatment. *Food Chemistry, 337*, 127973. 10.1016/j.foodchem.2020.127973 10.1016/j.foodchem.2020.127973 32927224

[CR70] Xu, Y., Wang, R., Zhao, H., Yin, Y., Li, X., Yi, S., & Li, J. (2020). Effect of heat treatment duration on the interaction between fish myosin and selected flavor compounds. *Journal of the Science of Food and Agriculture, 100*(12), 4457–4463. 10.1002/jsfa.10486 10.1002/jsfa.10486 32399966

[CR71] Yang Z-H, Emma-Okon B, Remaley AT (2016). Dietary marine-derived long-chain monounsaturated fatty acids and cardiovascular disease risk: A mini review. Lipids in Health and Disease.

[CR72] Yang Z-H, Miyahara H, Mori T, Doisaki N, Hatanaka A (2011). Beneficial effects of dietary fish-oil-derived monounsaturated fatty acids on metabolic syndrome risk factors and insulin resistance in mice. Journal of Agricultural and Food Chemistry.

[CR73] Young, A., & Dunn, E. (1975). *Report on a pilot study of fish silage*: North of Scotland College of Agriculture.

[CR74] Zamora R, Gallardo E, Hidalgo FJ (2007). Strecker degradation of phenylalanine initiated by 2, 4-decadienal or methyl 13-oxooctadeca-9, 11-dienoate in model systems. Journal of Agricultural and Food Chemistry.

[CR75] Zou T-B, He T-P, Li H-B, Tang H-W, Xia E-Q (2016). The structure-activity relationship of the antioxidant peptides from natural proteins. Molecules.

