# Supplementary material for: Pilot-Scale Ensilaging of Herring Filleting Co-Products and Subsequent Separation of Fish Oil and Protein Hydrolysates
Source: Food Bioproc Tech. 2022 Jul 19;15(10):2267–81. doi: 10.1007/s11947-022-02870-9 (PMC9295090; doi:10.1007/s11947-022-02870-9)
Supplement: Supplementary file 1 — Supplementary file1 (DOCX 630 KB) [file 11947_2022_2870_MOESM1_ESM.docx]

**Supporting information**

**Pilot-scale ensilaging of herring filleting co-products and subsequent separation of fish oil and protein hydrolysates**

Mursalin Sajib*, João P. Trigo, Mehdi Abdollahi, and Ingrid Undeland*

Food and Nutrition Science, Department of Biology and Biological Engineering, Chalmers University of Technology, SE-41296 Gothenburg, Sweden

*Corresponding Authors: E-mail: mursalin@chalmers.se; undeland@chalmers.se, Phone: +46317723820

Table 1. pH and temperature of silage during its pilot-scale production

| Sampling time | pH | Temperature (°C) |
| --- | --- | --- |
| 1 hour | 3.68 | 22.00 |
| 1 day | 3.74 | 21.00 |
| 2 days | 3.74 | 21.00 |

Table 2. Experimental plan for the separation of silage in a lab-scale batch centrifuge (20 °C; n = 4)

| g-force | Heat-treatment (85 °C; 30 min) | Centrifugation time (min) | | |
| --- | --- | --- | --- | --- |
| 3000 x g | Non-heat | 2 | 10 | 20 |
|  | Heat |  |  |  |
| 4500 x g | Non-heat | 2 | 10 | 20 |
|  | Heat |  |  |  |
| 8500 x g | Non-heat | 2 | 10 | 20 |
|  | Heat |  |  |  |

Table 3. Recovery yield of phases after centrifugation of non-heat-treated/heat-treated silage at different times and g-forces. Data are shown as mean values ± SEM (n=4).

|  |  |  | Yield (%; w/w) | | | |
| --- | --- | --- | --- | --- | --- | --- |
| g-force | **Time** | **Treatment** | **Oil (%)** | **Emulsion (%)** | **Hydrolysates (%)** | **Solids (%)** |
| 3000 | 2 | Non-heat | 1.26 ± 0.06 | 12.38 ± 1.60 | 1.73 ± 0.23 | 84.63 ± 1.70 |
|  |  | Heat | 5.73 ± 0.68 | 19.94 ± 1.36 | 9.42 ± 0.38 | 64.91 ± 1.60 |
|  | 10 | Non-heat | 0.36 ± 0.03 | 14.93 ± 0.81 | 27.23 ± 0.38 | 57.49 ± 0.90 |
|  |  | Heat | 5.54 ± 0.18 | 15.74 ± 0.78 | 38.22 ± 0.90 | 40.50 ± 1.16 |
|  | 20 | Non-heat | 0.65 ± 0.04 | 14.48 ± 0.60 | 34.57 ± 0.26 | 50.30 ± 0.69 |
|  |  | Heat | 6.52 ± 0.18 | 14.74 ± 0.73 | 40.52 ± 0.51 | 38.22 ± 0.64 |
| 4500 | 2 | Non-heat | 0.10 ± 0.01 | 33.11 ± 1.28 | 9.38 ± 0.33 | 57.41 ± 1.50 |
|  |  | Heat | 4.87 ± 0.97 | 25.14 ± 1.45 | 29.41 ± 0.37 | 40.58 ± 1.52 |
|  | 10 | Non-heat | 0.46 ± 0.03 | 23.16 ± 0.53 | 33.05 ± 0.21 | 43.33 ± 0.39 |
|  |  | Heat | 6.55 ± 0.99 | 12.09 ± 0.60 | 45.40 ± 0.99 | 35.96 ± 0.80 |
|  | 20 | Non-heat | 2.09 ± 0.09 | 16.05 ± 1.99 | 40.20 ± 0.34 | 41.66 ± 1.93 |
|  |  | Heat | 8.26 ± 0.36 | 10.45 ± 1.04 | 47.12 ± 0.51 | 34.17 ± 0.69 |
| 8500 | 2 | Non-heat | 5.85 ± 0.83 | 9.65 ± 1.30 | 17.86 ± 0.29 | 66.64 ± 0.80 |
|  |  | Heat | 9.03 ± 0.12 | 9.50 ± 0.30 | 42.11 ± 0.65 | 39.36 ± 0.43 |
|  | 10 | Non-heat | 3.75 ± 0.12 | 11.74 ± 1.50 | 42.12 ± 0.94 | 42.39 ± 0.64 |
|  |  | Heat | 9.50 ± 0.17 | 5.98 ± 0.72 | 51.68 ± 0.51 | 32.84 ± 1.02 |
|  | 20 | Non-heat | 4.14 ± 0.05 | 9.16 ± 1.12 | 48.15 ± 0.15 | 38.56 ± 1.11 |
|  |  | Heat | 9.72 ± 0.03 | 5.88 ± 0.57 | 53.06 ± 0.22 | 31.35 ± 0.34 |

Table 4. Color of oils recovered from heat-treated and non-heat-treated herring silage. Data are shown as mean values ± SEM (n=3).

| Origin | L* values | a* values | b* values |
| --- | --- | --- | --- |
| Non-heat-treated silage | 50.32 ± 0.02 | 2.62 ± 0.03 | 30.12 ± 0.02 |
| Heat-treated silage | 46.55 ± 0.08 | 0.36 ± 0.0 | 34.33 ± 0.11 |


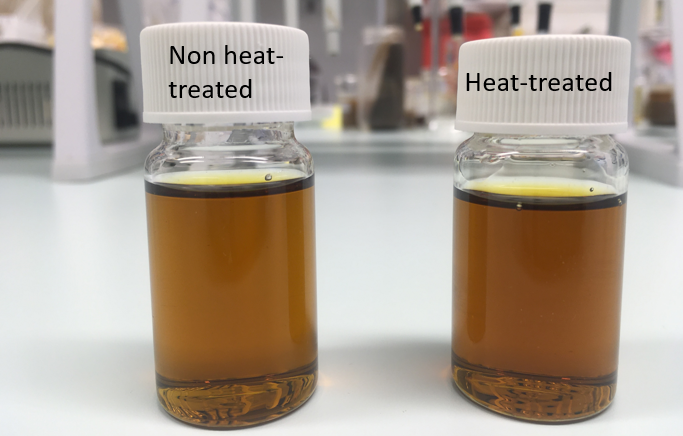


Figure 1. Herring oils recovered from herring filleting co-product silage
